# Consideration of health inequalities in systematic reviews: a mapping review of guidance

**DOI:** 10.1186/s13643-016-0379-1

**Published:** 2016-11-28

**Authors:** Michelle Maden

**Affiliations:** Department of Health Services Research, University of Liverpool, Liverpool Reviews and Implementation Group (LRIG), Second Floor, Whelan Building, The Quadrangle, Brownlow Hill, Liverpool, L69 3GB UK

**Keywords:** Health inequalities, Health equity, Systematic reviews, Guidance, Mapping review

## Abstract

**Background:**

Given that we know that interventions shown to be effective in improving the health of a population may actually widen the health inequalities gap while others reduce it, it is imperative that all systematic reviewers consider how the findings of their reviews may impact (reduce or increase) on the health inequality gap. This study reviewed existing guidance on incorporating considerations of health inequalities in systematic reviews in order to examine the extent to which they can help reviewers to incorporate such issues.

**Methods:**

A mapping review was undertaken to identify guidance documents that purported to inform reviewers on whether and how to incorporate considerations of health inequalities. Searches were undertaken in Medline, CINAHL and The Cochrane Library Methodology Register. Review guidance manuals prepared by international organisations engaged in undertaking systematic reviews, and their associated websites were scanned. Studies were included if they provided an overview or discussed the development and testing of guidance for dealing with the incorporation of considerations of health inequalities in evidence synthesis. Results are summarised in narrative and tabular forms.

**Results:**

Twenty guidance documents published between 2009 and 2016 were included. Guidance has been produced to inform considerations of health inequalities at different stages of the systematic review process. The Campbell and Cochrane Equity Group have been instrumental in developing and promoting such guidance. Definitions of *health inequalities* and *guidance* differed across the included studies. All but one guidance document were transparent in their method of production. Formal methods of evaluation were reported for six guidance documents. Most of the guidance was operationalised in the form of examples taken from published systematic reviews. The number of guidance items to operationalise ranges from 3 up to 26 with a considerable overlap noted.

**Conclusions:**

Adhering to the guidance will require more work for the reviewers. It requires a deeper understanding of how reviewers can operationalise the guidance taking into consideration the barriers and facilitators involved. This has implications not only for understanding the usefulness and burden of the guidance but also for the uptake of guidance and its ultimate goal of improving health inequalities considerations in systematic reviews.

## Background

Health inequalities are avoidable and unjust differences in health between individuals or populations [1]. Given that we know that interventions shown to be effective in improving the health of a population may actually widen the health inequalities gap while others reduce it, it is imperative that systematic reviewers consider how the findings of their reviews may impact (reduce or increase) the health inequality gap [2–4]. Furthermore, the existence of social inequalities, defined as “systematic differences in health between different socioeconomic groups within a society” (1, p. 473, [5]), increases the argument for *all reviewers*, not just those with a focus on health inequalities (HI), to consider the *potential* for their findings to reduce or increase HI. This is echoed by Cochrane and Campbell Collaborations who have called for the effects of interventions on HI to be considered by systematic review authors [6, 7].

Incorporating considerations of how review findings impact on health inequalities also aims to overcome one of the major barriers in using systematic reviews to inform decision-making, policy-making and practice [8]. Moving away from simply assessing what works to considering how review findings impact on disadvantaged populations, by assessing for example differential effects by subgroup populations can improve the applicability of the review findings to the local population[9, 10] thus increasing their *fit for purpose* in supporting decision-making and practice. Going beyond simply *does it work* to examine under what circumstances it works for whom and why [11] holds even more resonance when considering the impact on HI. As O’Neill et al. ([12], p. 57) point out, “the intervention has to be accessible, acceptable, effective in, and used by the most disadvantaged group within that population to be truly effective at reducing inequities in health” (whilst health inequalities are defined as avoidable differences in health outcomes across individuals or between populations, the narrower but related term health equity is often referred to in the literature as health inequalities which are also considered *unfair and unjust* [13].)

Methodological research has highlighted an absence of evidence with regards to the extent to which systematic reviews taking into account issues of HI when analysing and making recommendations for further research and practice [3, 14, 15]. Furthermore, recent methodological studies of systematic reviews demonstrated that very few (<5%) addressed differential impacts across socio-economic groups [16, 17]. The extent to which systematic reviewers in the past have failed to consider how their review findings impact on HI, is due in part to the focus reviewers placed on the *effectiveness* of interventions and also due to a lack of relevant data reported in the primary literature to assess such differential effects [18]. In addition, the lack of guidance or awareness of the existence of such guidance in this area may also have worsened the situation. More importantly, however, it is also due to a failure on the part of review authors to even consider differential impacts in reviews where HI is not the focus [8, 17, 19].

This paper aims to review existing guidance on incorporating considerations of HI in systematic reviews to examine the extent to which they can help reviewers incorporate such considerations in systematic reviews.

## Aim and objectives

The aim of this study was to undertake a mapping review of existing guidance documents currently provided to assist reviewers when determining whether and how to incorporate considerations of HI. A mapping review aims to map out and categorise the literature according to key features (e.g. study design) on a particular topic and to identify gaps in the research literature [20]. The objectives were (1) to provide an overview on the types of guidance, in particular the focus, scope and purpose of the guidance, (2) to explore how the guidance is defined by authors, (3) to describe the methods used to develop the guidance, (4) to examine the comprehensiveness, overlap and operationalisation of the guidance.

## Search strategy

A systematic approach to identifying the literature was undertaken in a two-tiered approach. Firstly, more generic guidelines to evidence synthesis were located and searched to identify specific guidance relating to the incorporation of HI. A search of review guidance manuals prepared by international organisations engaged in undertaking evidence synthesis was undertaken (see Table 1). Publications listed on the Campbell and Cochrane Equity Group website were also scanned.

**Table 1:** International evidence synthesis organisation websites

Agency for Healthcare Research and Quality (www.ahrq.gov) The Cochrane Collaboration (www.cochrane.org) The Campbell and Cochrane Equity Methods Group (http://methods.cochrane.org/equity/) Centre for Reviews and Dissemination at the University of York (http://www.york.ac.uk/crd/) EPPI-Centre (http://eppi.ioe.ac.uk/) Health Technology Assessments (http://www.nets.nihr.ac.uk/programmes/hta) European Network for Health Technology Assessments (EUnetHTA – http://www.eunethta.eu/) National Institute for Health and Care Excellence (NICE – www.nice.org.uk) Joanna Briggs (http://joannabriggs.org/sumari.html)	

In addition to this, a search was undertaken in Medline, CINAHL and The Cochrane Methodology Register. Key terms searched on included thesaurus and textwords terms comprising of synonyms for health inequalities, evidence synthesis and methodology/guidance/tools (see Appendix 1). A pre-published search strategy designed to capture HI studies was reviewed and utilised [21]. A practical approach to developing a search strategy to identify different types of evidence synthesis was adopted. This approach was informed by published systematic review filters and related evidence synthesis terms (e.g. realist review, realist synthesis, integrative). Searches were undertaken in September 2015, and search alerts were set up to capture relevant articles added into the databases after this date. No restrictions by year were applied, but publications were limited to English language only studies.

Requests for guidance were made via relevant email discussion lists and contacting experts in the Campbell and Cochrane Equity Group. For guidance that had been updated, the most recent update was included. Where multiple publications discuss the same guidance they were considered together.

## Inclusion criteria

Any type of study was included if it provided an overview or discussed the development and testing of a conceptual or practical framework, tool, model or guidance (advice or formal recommendations) for dealing with the incorporation of considerations of HI in evidence synthesis. For the purpose of this review, HI is defined according to Whitehead’s ([1], p. 473) definition in which *inequalities* in the British context—and increasingly also across Europe—carries the same connotations of unfairness and injustice as the term *inequities*. Both generic guidance (e.g. guidelines published by evidence synthesis organisations or collaborations) that incorporated considerations of HI and HI-specific guidance (e.g. scholarly methodological studies presenting specific guidance for incorporating considerations of HI in systematic reviews) were included. Studies that primarily offer a theoretical discussion or comments on if and why HI should be included in evidence synthesis, or frameworks and guidance for the incorporation of HI for purposes other than evidence synthesis, were excluded. For practical reasons, studies were limited to English language publications. Screening of studies was undertaken by the author.

## Data extraction

Data were extracted by the author on targeted audience (e.g. for reviewers, users of evidence synthesis), purpose (e.g. tools are to be used for planning, conducting, reporting, disseminating or using systematic reviews), scope (e.g. to inform systematic reviews, other research), how the tool was developed, operationalisation of the tool (how reviewers are instructed to apply the guidance) and whether and how they define health inequalities*.* Included studies were also categorised as to their focus, i.e. whether they were HI, or had a generic focus but which accounted for HI.

## Data synthesis

Results are summarised in narrative and tabular forms. Strengths and weaknesses of the guidance in assisting systematic reviewers were assessed based on methods of development including evaluation, accessibility and transparency in operationalisation.

## Results

**Fig. 1:**
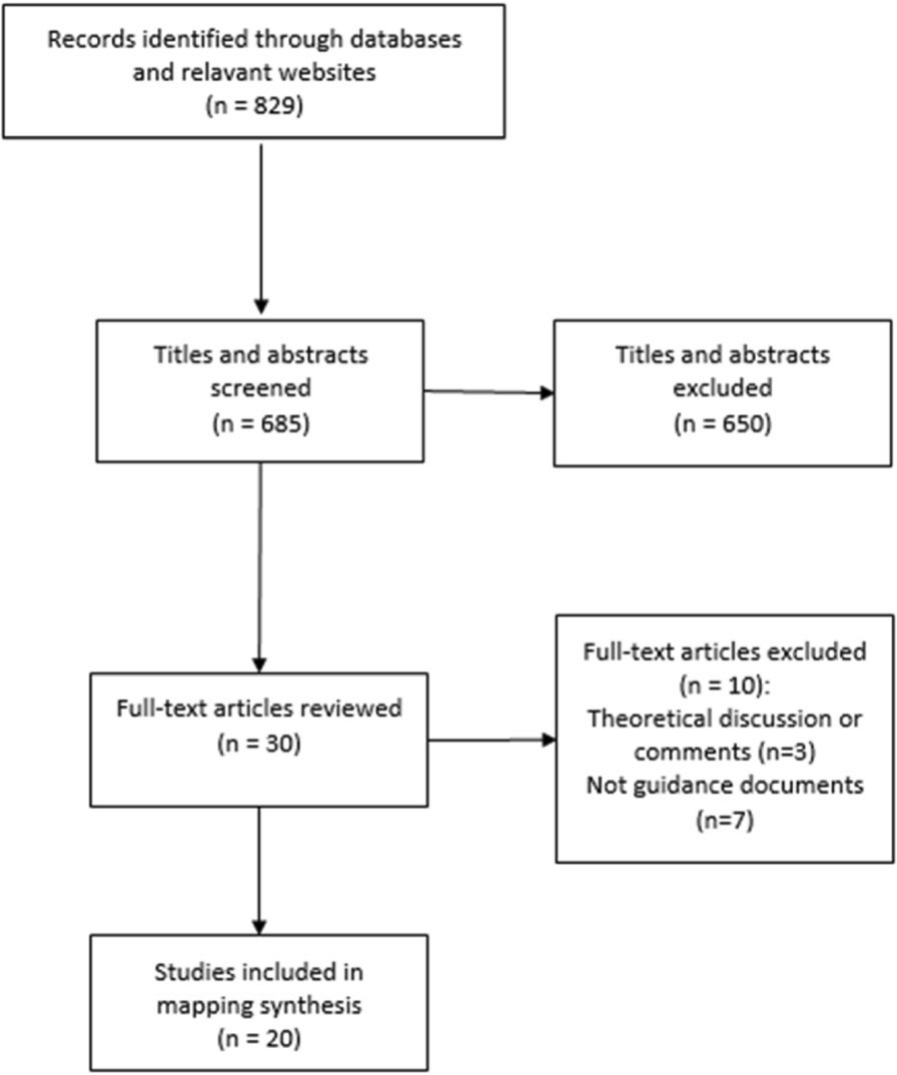
Flow of search results

The results of the search are summarised in Fig. 1. Eight hundred and thirty-six references were identified of which 20 were included in the review. All guidance documents [12, 22–40] incorporating considerations of HI in systematic reviews were published between 2009 and 2016. Table 2 outlines the characteristics of the included studies. Doull et al. [22] report on three guidance documents within the same study [23–25]. Tugwell et al. [29] and Ueffing et al. [30] both discuss the Cochrane Equity Checklist, whilst Welch et al. [32, 33] and Burford et al. [34] report on the PRISMA-Equity 2012 Extension. The majority of the guidance has been produced with the involvement of members of the Campbell and Cochrane Equity Methods Group.

**Table 2:** Characteristics of included studies

Guidance	Focus		Purpose			Scope					Place of publication			Open access	HI defined
	Equity	Generic	Systematic Review	Intervention studies	Other	Planning	Conduct	Reporting	Applicability	Knowledge Translation	Article	Book Chapter	Online only		
Armstrong et al. [37]^a^		✓	✓				✓					✓		✓	✓
Armstrong et al. [38]^a^		✓	✓				✓					✓		✓	✓
Chambers and Wilson [39]		✓	✓						✓			✓		✓	
CRD [40]		✓	✓				✓					✓		✓	
Doull et al. [22]^b^	✓^e^		✓			✓	✓				✓			✓	✓
Doull et al. (25)^b^	✓^e^		✓			✓	✓						✓	✓	✓
Puil et al. [24]^b^	✓^e^		✓			✓	✓						✓	✓	✓
Welch et al. [23]^b^	✓^e^		✓			✓	✓				✓			✓	✓
NIHR CLAHRC North West Coast [26]	✓^f^		✓	✓	✓^g^	✓							✓	✓	✓
Nasser et al. [27]	✓		✓			✓					✓				✓
O’Neill et al. [12]	✓		✓	✓		✓	✓	✓			✓				✓
Oxman et al. [28]	✓		✓						✓		✓			✓	✓
Tugwell et al. [29]^c^	✓		✓				✓	✓			✓			✓	✓
Ueffing E et al. [30]^c^	✓		✓			✓	✓						✓	✓	✓
Tugwell et al. [31]	✓		✓							✓	✓			✓	
Welch et al. [32]^d^	✓		✓				✓	✓			✓			✓	✓
Welch et al. [33]^d^	✓		✓				✓	✓			✓			✓	✓
Burford et al. [34]^d^	✓		✓				✓	✓			✓			✓	✓
Welch et al. [35]	✓^e,f^		✓			✓					✓			✓	✓^h^
Welch et al. [36]	✓		✓				✓			✓	✓			✓	✓

^a^Guidance from the Cochrane Collaboration

^b^Doull et al. [22] report on three guidance documents [23–25] within the same study

^c^Report on the Cochrane Equity Checklist [29]

^d^Report on the PRISMA-Equity 2012 Extension [32]

^e^Sex and gender focus

^f^Socioeconomic focus

^g^Applied research, evidence synthesis, capacity building and knowledge exchange and implementation

^h^HI defined, SES/sex and gender not defined

## Focus, scope and purpose of guidance

Table 2 outlines the focus, scope and purpose of the guidance. The majority of the guidance documents have an HI focus; three focus on sex and gender [23–25], one on socio-economic status (SES) [26] and one on both sex and SES analysis in systematic reviews [35] All four generic guidance documents in which HI was considered, signposted reviewers onto HI-focused guidance produced by the Campbell and Cochrane Equity Group or the PROGRESS framework [12]. All guidance were produced for informing the production of systematic reviews with two guidance documents also applicable to other types of research [12, 26]. Guidance has been produced to inform considerations of HI at different stages of the systematic review process with the most guidance produced to support the conduct of systematic reviews.

## Guidance definitions

The documents defined their guidance in different ways, for example, as briefing notes [22–25] equity lens [12, 26, 27, 29, 30], recommendations [29, 30, 36], plausibility algorithm [35], tool [28] framework [31, 39], guidance [37, 38, 40] and guidelines [32–34].

All but three guidance documents [31, 39, 40] define what is meant by HI, equity or inequity. Where definitions are recorded, they differed across the studies. Whitehead’s [13] definition of health equity and HI were the most commonly reported within the guidance documents, although a number of different authors were cited for HI [8, 13, 41, 42], health inequity [13, 41, 43] and health equity [13, 44–48]. Four of the five guidance documents with a sex and gender HI focus all define what it meant by sex and gender in the same way [22–25]. Neither of the two guidance documents with a socio-economic focus define socio-economic status [26, 35].

## Development of guidance

Table 3 outlines the guidance development process of the included studies. The majority of the guidance documents were transparent in outlining how the guidance had been produced. Of those reporting methods of guidance development, all were informed by a literature review.

**Table 3:** Development of guidance

Guidance	Aim/audience	Development method	Operationalisation	Strengths/limitations
HI-focused guidance				
Doull et al. [22] Welch V., et al. for the sex/gender methods group. [23] Puil L., et al. for the sex/gender methods group. [24] Doull M., et al. for the sex/gender methods group. [25]	To translate knowledge about sex/gender analysis into a user-friendly briefing note format and evaluate its use in aiding in the implementation of sex/gender analysis in systematic reviews. Aimed at reviewers and editors of Cochrane Aids/ Hypertension and Musculoskeletal Groups	Guidance development process • Informed by literature reviews •Built on existing structured guidance for systematic reviewers • Feedback and revision sought • Evaluated by 19 participants attending a workshop at the 2012 Canadian Cochrane Symposium • Underpinned by diffusion of innovations theory Who else was involved: Members of the Cochrane Collaboration HIV/AIDS, Hypertension, and Musculoskeletal Review Groups with expertise in methodology, sex/gender analysis, systematic reviews, policy and knowledge translation and additional clinical experts	Four sections: Sections 1–3 define the issue, definitions and rationale for considering sex and gender analysis. Section 4 has 13 items to consider in relation to sex and gender (question formulation; context; population; intervention/ comparator; outcomes; study design; searching for studies; data collection; risk of bias; data analysis; additional analyses; presenting results and summary of findings; interpreting and drawing conclusions). Topic specific descriptive examples provided	Strengths: • Wide range of expertise and systematic review experience involved in development • Consensus-based •provides rationale, evidence and examples to operationalise guidance • Piloted and evaluated Open access Limitations: • Evaluated by a self-selecting group attending Cochrane Meeting. •Terminology used (logic model, context) may not be widely accepted or understood
NIHR CLAHRC North West Coast [26]	To help ensure that all activities of the NIHR CLAHRC NWC have potential to contribute to reducing health inequalities. Aimed specifically at anyone undertaking CLAHRC NWC work (including reviewers) but also anyone wishing to consider HI in their research	Guidance development process: Collaborative process in a series of workshops in 2014/2015 Who was involved: NIHR CLAHRC NWC staff and partners	Four sections: 1. Clarifying the health inequality dimensions of the problem 2. Designing your intervention/action 3. Evaluating and/or monitoring the impact of your activity 4. Planning for wider impacts on health inequalities Included 26 questions Each section also includes a Health Inequalities Assessment of an exemplar proposal for applied research. Links to resources that provide more information about the issues covered in each section. Guidance provided on how to use HIAT.	Strengths: • Revised after feedback from users, plan to revise regularly after further user feedback. encourages involvement of the public/team approach in considering equity in reviews. •Addition of further resources •Worked example provided •open access Limitations: • Limited information on how the guidance was developed or tested. • Long checklist • Worked example is not a systematic review therefore further details on *how* reviewers can operationalise individual items is required
O’Neill et al. [12]	To assess the utility of an acronym, place of residence, race/ ethnicity/culture/language, occupation, gender/ sex, religion, education, socioeconomic status, and social capital (“PROGRESS”) to guide the conceptualization of disadvantage, data extraction, and to inform equity analyses in systematic reviews. Aimed at reviewers, researchers and users	Authors demonstrate how an existing framework PROGRESS, the framework for the PRISMA Equity Extension, can be applied to systematic reviews	Asks reviewers to consider variations in health across 8 factors: place of residence, race/ethnicity/culture/ language, occupation, gender/sex, religion, education, socioeconomic status and social capital. For each PROGRESS factor, examples are provided that demonstrate differences in burden of disease and an effective intervention that could reduce that burden.	Strengths: • Considers multiple equity dimensions Limitations: • Limited examples provided further detail on how reviewers can operationalise individual items is required • Not evaluated • Not open access
Nasser et al. [27]	To develop and pilot an equity lens to help researchers develop a more equity-oriented approach toward priority setting and agenda setting in systematic reviews Aimed at reviewers.	Development process • A workshop presenting survey results from a previous project • Literature review • Workshop for refinement of the equity lens • Piloted • Underpinned by conceptual framework for priority setting Who was involved: 15 people attending the 2008 Cochrane Colloquium attended the first workshop, 12 attending the 2009 Cochrane Collaboration attended the second workshop	Two checklists: 1. 9 questions assessing priority setting, from identifying the questions and stakeholders to the evaluation strategy. 2. 8 questions assessing the outcome evaluation of priority setting	Strengths: • Piloted Limitations: • Evaluated by a self-selecting group attending Cochrane Meeting • Not open access
Oxman et al. [28]	To present a structured approach to considering the impacts of policy and programme options on inequities, to inform decisions about what options to implement and how to implement them. Aimed at users	Not reported	4 questions that can be used to guide considerations when using systematic reviews regarding impacts on inequities.	Strengths: • Descriptive examples provided • Open access Limitations: • No information available on how the guidance was developed or evaluated. • Terminology used may not be widely accepted or understood • Greater detail required on *how* reviewers can operationalise the items
Tugwell et al. [29] Ueffing E, et al. for the Campbell and Cochrane Equity Methods Group. [30]	To provide guidance on assessing equity for users and authors of systematic reviews of interventions. Aimed at reviewers, users and journal editors.	Development process: • 4 working sessions • Built on previous work by the members of the Measurement and Evidence Knowledge Network • Panel members reviewed the evidence and drafted guidance • Feedback and revision sought Who was involved: International leaders in systematic reviews and health equity, mixed methods experts, social scientists, economists, experts in systematic reviews, experts in public health and health equity, experts from low and middle income countries and policy advisers who use systematic reviews. Members of the Campbell and Cochrane Equity Methods Group and the Measurement and Evidence Knowledge Network	7 recommendations underpinned by 16 checklist items. Examples provided	Strengths: • Wide range of expertise involved in development • Consensus-based •Descriptive examples provided •Addition of resources to signpost reviewers to sources of help when attempting to answer the questions. Limitations: • Terminology used may not be widely accepted or understood • Greater detail required on *how* reviewers can operationalise the items
Tugwell et al. [31]	“Propose an evidence based framework – or “cascade” – for equity-orientated knowledge translation.” Aimed at reviewers, researchers and users	Development process: Not reported Who was involved: Not reported	5 steps Examples demonstrate how the steps are applied to 2 systematic reviews	Strengths: • Descriptive examples provided to operationalise items • Open access Limitations: • No information available on how the guidance was developed or tested. • Does not define equity
Welch et al. [32] Welch et al. [33] Burford et al. [34]	“To provide structured guidance on transparently reporting methods and results for equity focused reviews. To legitimise and emphasize the importance of reporting health equity results.” Aimed at reviewers	Development process: • Consensus-based - Followed guidance for developing reporting guidelines • Identifying need • Reviewing the literature (systematic review and methodological study) • Gathering expert opinion (online survey) • Exploring consensus • Piloting Who was involved: • Equity researchers, decision-makers, clinical epidemiologists, systematic review methodologists, journal editors, funders, practitioners, review authors with LMIC focus, methodologists/statisticians, novice systematic reviewers and established systematic reviewers involved with equity and/or complex population intervention systematic reviews	14-item equity extension of existing guidance for the reporting of systematic reviews. Provides detailed rationale, evidence, whenever available, an exemplar for recommending each item and examples of good practice.	Strengths: • Wide range of expertise involved in development • Involved non-expert reviewers in development • Consensus-based • Followed guidance on developing reporting guidelines • Provides rationale, evidence, exemplars and examples to operationalise items • Evaluated • Open access Limitations: • Terminology used may not be widely accepted or understood • Greater detail required on *how* reviewers can operationalise some items, e.g. approach to logic model
Welch et al. [35]	To develop and assess inter-rater agreement for an algorithm for systematic review authors to predict whether differences in effect measures are likely for disadvantaged populations relative to advantaged populations. Aimed at reviewers.	Development process: • Follows established methods of checklist development • Review of existing guidance • Systematic review of methods for assessing effects on health equity • Survey of practitioners/managers • Evaluated face and conceptual validity amongst four clinical methodologists • Inter-rater reliability assessed amongst 35 methodologists, clinicians, users of SRs assessed the algorithm against a pre-selected sample of 10 SRs. • Piloted Who was involved: Authors, practitioners/managers, clinicians, methodologists, users, members of Cochrane Collaboration	3 questions. Examples operationalise how each of the questions may result in differential effects	Strengths: • Wide range of expertise involved in development • Follows established methods of checklist development • Descriptive examples provided to operationalise items • Evaluated • Open access Limitations: • Low inter-rater reliability • Tested by individuals rather than review teams who evaluated the algorithm against summarised information from the reviews • Subject expertise of the raters is unclear, this may have impacted on whether they would anticipate differential effects. • Multi-component questions cover several factors
Welch et al. [36]	To provide guidance on how to conduct equity-focused systematic reviews consistent with the recommendations of PRISMA-E 2012 to facilitate the use of both guidance documents. This article also discusses challenges related to knowledge translation for equity-focused systematic reviews. Aimed at reviewers.	Development process: • Series of methodology meetings • Systematic review of methods to assess equity in systematic reviews • Methods study • WHO Task force on evidence-informed policies about health systems • PRISMA-Equity (2012) guidance Who was involved:Campbell and Cochrane Equity Methods Group, Cochrane Public Health Review Group, methodologists, funders, journal editors, clinicians and public health practitioners	10 steps to considering health equity in reviews. Recommendations with a few brief examples from exemplar reviews	Strengths: • Wide range of expertise involved in development • Descriptive examples provided to operationalise items • Open access Limitations: • Terminology used may not be widely accepted or understood • Greater detail required on *how* reviewers can operationalise the items
Generic focused guidance				
Armstrong R, Waters E, Doyle J (editors) [37] Chapter 21: Reviews in health promotion and public health. In Higgins JPT, Green S (editors). Cochrane Handbook for Systematic Reviews of Interventions Version 5.1.0. Rebecca Armstrong, Elizabeth Waters on behalf of the Guidelines for Systematic Reviews in Health Promotion and Public Health Taskforce. [38] Systematic Reviews of health promotion and public health Interventions.	Guidance to authors for the preparation of Cochrane Intervention reviews (including Cochrane Overviews of reviews). Aimed at reviewers.	Not reported	N/A^a^	
Chambers and Wilson [39]	To enable researchers to present and contextualize evidence from systematic reviews and other sources of synthesized and quality-assessed evidence. Aimed at researchers.	Uses the Oxman et al. [28] criteria	4^b^	Strengths: • As above for Oxman et al. [28] • Authors offer advice on operationalising guidance in absence of evidence in reviews, “by information gathered locally, using documents produced by or relevant to the NHS, such as Joint Strategic Needs Assessments and equity audits.” Limitations: As above for Oxman et al. [28]
CRD [40]	To promote high standards in commissioning and conduct, by providing practical guidance for undertaking systematic reviews evaluating the effects of health interventions. Aimed at reviewers.	Not reported	N/A^c^	

^a^Signposts reviewers to The Campbell and Cochrane Equity Methods Group

^b^Follows tools developed by SUPPORT collaboration [28]

^c^Signposts reviewers to PROGRESS and The Campbell and Cochrane Equity Methods Group

The guidance development process for most involved seeking feedback and revision from people with a wide range of expertise (including researchers, HI experts, review methodologists, decision-makers, clinical epidemiologists, practitioners and journal editors) and systematic review experience, the majority of whom were either members of the Cochrane Collaboration or were attending Cochrane Workshops. Burford et al. [34] and Doull et al. [22] specifically report involving novice reviewers in the development of their guidance. The PRISMA-E 2012 reporting guidelines [32, 33] were also informed by consensus methods. Whilst all of the guidance documents were produced from an HI perspective, only five had theoretical underpinnings or followed established methods in developing their guidance [22, 27, 32, 33, 35].

## Operationalisation of guidance

As a means of demonstrating *what* reviewers should consider in the application of the items, most of the guidance provide examples from published systematic reviews. For example, when asking reviewers to consider whether there are known or possible differences by sex/gender, Welch et al. [23] use the following example, “In a systematic review on quality of life after total hip and total knee arthroplasty, men appeared to benefit more from the intervention in the few studies that addressed this issue.”

Despite the different purposes and audiences, there is a considerable overlap in what users of the guidance are asked to consider at different stages of the review process. Table 4 highlights this using the example of whether to expect differential effects across SES population characteristics.

**Table 4:** Overlap of guidance items on anticipating differential effects across SES in relation to population characteristics

Guidance	Purpose	Item (i.e. what reviewers are asked to consider)
*Can we expect differential effects across socioeconomic status in relation to population characteristics?*		
Welch et al. [36, p. 2]	Conduct Knowledge translation	*Define conceptual approach to health equity* “whether social gradients exist in the burden of disease and whether relative or absolute effects of interventions are likely to differ for disadvantaged populations” *Frame the health equity question* “This requires consideration of both relative risk and absolute effects, as well as baseline risk of the health outcome of interest across social gradients.”
Welch et al. [35]	Planning	“Are there differences in patient/community/population characteristics (e.g. underlying pathophysiology, comorbidities, patient attitudes, etc.) that are likely to create important differences in the magnitude of relative effect of the intervention versus the control for the outcome of interest?”
Oxman et al. [28]	Applicability	“Which groups or settings are likely to be disadvantaged in relation to the option being considered?” “Are there plausible reasons for anticipating differences in the relative effectiveness of the option for disadvantaged groups or settings?” “Are there likely to be different baseline conditions across groups or settings such that that the absolute effectiveness of the option would be different, and the problem more or less important, for disadvantaged groups or settings?”
NIHR CLAHRC North West Coast [26]	Planning	“What evidence is there that this problem is unequally distributed across socio-economic groups?” “What aspects of socio-economic inequalities can be expected to impact on this problem?”
Welch et al. [23]	Planning Conduct	*Question formulation*: “Consider whether there are known or possible differences by sex/gender across: baseline risk, prevalence, vulnerability, implementation or response to intervention, and plan objectives and methods accordingly.”

The rationale for *why* HI should be considered was also provided in some guidance. For example, Oxman et al. ([28], “Questions to consider”, no.3) ask “are there likely to be different baseline conditions across groups or settings such that the absolute effectiveness of the option would be different, and the problem more or less important, for disadvantaged groups or settings?” They then outline the rationale, “Typically, baseline risks are larger in disadvantaged populations and a larger absolute effect could therefore be expected.” ([28], “Questions to consider”, no.3).

When addressing *how* reviewers can operationalise the items, the comprehensiveness and application with which this is detailed differs across the guidance. For example, the number of items reviewers are asked to consider in the guidance documents ranges from 3 up to 26. Many guidance documents recommend using a theory-based approach, using programme theory or logic models to understand the assumptions behind how and why the intervention may work differently across disadvantaged populations and the influence of context on the outcome [22–26, 29, 30, 32, 33, 35, 36]. However, there is a lack of detail on how this could be implemented in practice. Tugwell et al. ([29], 2.“Defining disadvantage”, para. 2), for example, state that “implications on inequities are dependent on context, so authors of HI orientated reviews must strive to understand and explore the mediating effect of context”, yet they do not define what is meant by *context* and what data could be collected to explore this. Welch et al. [32] suggest that one limitation of the guidance is the use of terminology such as *logic model*, *analytic framework*, *context* and *process evaluation*, terms that are not widely accepted. A lack of consistency was noted in applying such terms across the guidance with some referring instead to *causal pathway analysis*, *program theory* and *mechanisms of action*.

Few guidance documents discuss who should be involved in making decisions on if and how HIs matter in systematic reviews. Tugwell et al. [29] suggest that relevant stakeholders should be involved in defining the review question, whilst HIAT [26] recommend involving members of the public (e.g. service users, carers, people living in disadvantaged neighbourhoods) in the planning stages. Only Welch et al. [35] started to explore how people came to their decisions of whether to expect differential effects across sex/SES and found that these decisions were made based on theory, personal experience, empirical data and guesses, but called for further research to investigate how these are used to make judgements.

## Evaluation of guidance

Formal methods of evaluation were reported in three studies [22, 34, 35] for six of the guidance documents [23–25, 32, 33, 35]. Burford et al. [34] surveyed 151 systematic review authors on the perceived utility of the PRISMA-E 2012 [32, 33]. Reported advantages of using PRISMA-E 2012 include improved awareness of HI considerations in systematic reviews and improved consistency of reporting of HI in systematic reviews. Barriers reported include time required to apply guidance, length of guidance, increase length and complexity of reviews and lack of data in primary studies to operationalise some of the guidance.

Doull et al. [22] undertook a workshop evaluation involving 19 participants including potential users (researchers, practitioners and policy-makers) with little or some knowledge of the concepts of sex/gender to evaluate the content, readability and comprehensiveness of their briefing notes [23–25]. Although respondents reported that the briefing notes “provided clear methodological guidance to address sex/gender in reviews” ([22], Results, para. 2) and rated all aspects highly, responses were mixed on the level of complexity within the methods section.

Finally, in Welch et al. [35] four clinical methodologists evaluated the face and construct validity of a plausibility algorithm in predicting the likelihood of differential effects across sex and SES. Thirty-five review users, methodologists and clinicians also assessed the inter-rater reliability of the algorithm against ten pre-selected systematic reviews. The results found a low to no agreement beyond chance between raters for each of the three questions across sex and socioeconomic considerations. The authors suggest several reasons for the poor agreement relating to the design of the algorithm (use of multi-component questions covering several factors, omission of a *don’t know* category for responses), individual characteristics of respondents and poor choice of proxy or gold standard set of reviews to test the algorithm against. Whether any of the guidance has resulted in an uptake of considerations of HI in systematic reviews is still to be determined [22, 33].

## Discussion

This review identified 20 guidance documents for incorporating considerations of equity in systematic reviews spanning the whole spectrum of the review process, from planning, conduct and reporting through to considerations of applicability to disadvantaged populations and knowledge translation when using reviews to inform decision-making and policy. Many of the documents have been published in the last 3 years, highlighting the fact that methodological research in this field is an important, emerging and evolving area of interest. The majority of the guidance is published in open access format in the journal literature. Whilst this increases the accessibility of the guidance, omission from standard review guidance handbooks places a greater emphasis on guidance authors to raise awareness of the existence of such tools to encourage greater uptake. For example, Welch et al. [32, 33] recognise the importance of widespread dissemination amongst journal editors, funding bodies and ethics committees in order to encourage the adoption of the PRISMA-Equity 2012 Extension reporting guideline.

Increasing awareness of such guidance is even more important when reviewers are faced with the different terminology used by authors to describe it. Use of multiple terms such as algorithm, equity lens, tool, may make them harder to locate within the journal literature. The Campbell and Cochrane Equity Methods Group have started to collate guidance on HI considerations for review authors [49], yet the list does not cover all guidance identified in this review and focuses to a greater extent on the guidance developed to support Cochrane Review authors.

Whitehead’s [13] definition of HI as “Inequality in health is a term commonly used in some countries to indicate systematic, avoidable and important differences” and health inequity as “refers to differences in health which are not only unnecessary and avoidable but, in addition, are considered unfair and unjust” were most commonly applied in the guidance. However, rather than demonstrate an acceptance of a common definition for HI or health equity, most of the guidance citing Whitehead [13] were produced by the same group (The Campbell and Cochrane Equity Methods Group). The different definitions of HI and health equity used by the guidance documents supports the view by Tugwell et al. [29] that these terms are used in different ways by different authors, and that there is no agreed definition of health equity or HI. This stresses the importance therefore for HI guidance to define health equity and/or HI to help reviewers to operationalise the guidance.

This is particularly true for guidance incorporating considerations of SES in systematic reviews. Neither of the two guidance documents with a focus on SES [26, 35] defined the term. Several of the guidance documents refer reviewers to the PROGRESS framework [12, 50] when asking reviewers to consider disadvantage. PROGRESS although does not explicitly define SES, relates it to income, while considering education and occupation separately. Yet SES has been defined more broadly as “a composite measure that typically incorporates economic status, measured by income, social status, measured by education; and work status measured by occupation” ([51], pp. 30), [52, 53]. The classification of individuals by SES has implications for reviewers in relation to types of SES indicators to collect and therefore, the definition and classification of HI terms such as SES needs to be operationalised within the guidance. This is further supported by the findings of Runnels et al. [54] who in a survey examining the challenges of including sex/gender analysis in systematic reviews found that one of the significant challenges was clarifying the concepts of sex and gender.

Much of the guidance is written from the perspective of HI having already being identified as the focus of the review and from a whole HI perspective rather than focusing on a specific HI dimension. More recently, however, guidance is being tailored more towards specific HI domains, in particular, sex/gender [23–25, 35] and SES [26, 35]. This may reflect the interests of the groups involved in producing the guidance. For example, HIAT [26] was developed by the National Institute of Health Research Collaboration for Leadership in Applied Health Research and Care North West Coast (NIHR CLAHRC NWC) whose remit is to ensure that all of the research it produces has socio-economic relation to HI as its core focus (www.hiat.org.uk).

Guidance on incorporating sex/gender analysis in systematic reviews was produced by the Cochrane Sex/Gender Methods Group (a subgroup of the Campbell and Cochrane Equity Methods Group). However, the development of more HI-specific guidance may also suggest that once reviewers have identified which HI domain(s) to consider, they may have difficulty operationalising the more generic HI-focused tools and require more tailored guidance. Or it may be that given that it would be impossible for reviewers to incorporate considerations across all HI dimensions, that there is a debate developing that certain HI domains may be considered more important than others to incorporate in systematic reviews. Indeed, in the application of the PROGRESS framework to systematic reviews, O’Neill et al. [12, p. 62] state that the framework is “not intended to encourage data dredging but to identify the most important factors that drive inequities in health”.

The Campbell and Cochrane Equity Group have been instrumental in driving forward methodological advances in evidence synthesis for the incorporation of HI in systematic reviews, and although not specifically stated, most of the guidance produced appears most relevant to reviewers undertaking systematic reviews measuring the effectiveness of interventions or at users assessing the applicability of reviews of effectiveness. This is no doubt partly due to the focus of the Cochrane Collaboration on reviews of effectiveness, yet how well this guidance translates to other types of HI systematic reviews (e.g. qualitative) therefore is unclear, and further research investigating the usefulness of the guidance for other types of reviews is required.

One potential challenge facing reviewers in operationalising the guidance is that methodological approaches to incorporating considerations of HI are still in development, and even the guidance authors themselves recognise that one of the limitations of the guidance may relate to the terminology used, such as logic model, process evaluation, mechanisms of action, terms which may not be accepted [32] or understood [55]. Encouraging reviewers to consider what works for disadvantaged populations, why, how and under what circumstances not only requires as Tugwell et al. ([29], Conclusions, para. 2) suggest “a paradigm shift in the generation and synthesis of evidence”, but also an acceptance of the terminology along with an understanding of *how* these methods can be applied in practice. Part of the problem is that current review methods cannot necessarily be transferred across to consider more complex issues and HI [3, 11] and methods to incorporate such complexity in systematic reviews are only just emerging [9, 11, 56–61]. Given the potential complexity of the process therefore, further research examining the challenges and barriers in incorporating considerations of HI in systematic reviews is required to better support reviewers in undertaking HI-focused reviews.

There is also perhaps, a greater need to understand how guidance items can be operationalised. Most of the guidance is operationalised by means of descriptive examples from published systematic reviews, and there is evidence to indicate that reviewers find them useful. For example, Doull et al., [22] in testing their guidance on incorporating issues of sex/gender analysis in systematic reviews found that reviewers wanted even more examples.

Much of the guidance is written with an underlying assumption that reviewers will recognise if and how equity matters. Empirical evidence however suggests otherwise [35, 54]. A recent survey by Runnels et al. [54] conducted on the challenges of including sex/gender analysis in systematic reviews seems to support this view. They found that concerns were raised over the construction of knowledge and cites one respondent saying “the biggest challenges are much more fundamental and have to do with the way that we arrive at decisions as to what is important for us to study, why it is important for us to study, and how we determine the way to study” ([53], Conceptual challenges, para. 3).

Welch et al., [35] in developing their equity plausibility algorithm, start to explore the rationale behind reviewers’ decisions and found that empirical data, theory or personal experience were often used to explain their reasons but call for further research to enhance understanding of how this was used and the contribution of individual characteristics to the process. In order to help reviewers to operationalise the guidance therefore, it may be useful to explore the rationale behind how reviewers are making decisions when applying the guidance and the contribution made to those decisions by different individuals (e.g. stakeholders).

Furthermore, if, as Welch et al. ([35], Discussion, para. 1) suggest, reviewers “need to have a deep understanding of the content area” to make judgements about likely differential effects, then single examples drawn from topic-specific reviews may not be the best way to demonstrate guidance application. Without a comprehensive understanding of the different ways in which HI issues may contribute towards differential effects in health outcomes, it may be difficult for some reviewers, particularly those new to HI, to recognise the need to incorporate or operationalise such issues in systematic reviews. Building on this, research is currently underway by the author (MM) to identify a comprehensive set of considerations to help reviewers operationalise the influence of socio-economic contextual factors on how and why an intervention may work differently across disadvantaged populations.

Strengths of the guidance evaluation methods include the involvement of a wide range of expertise (e.g. reviewers, methodologists, decision-makers, HI experts), consensus methods and piloting of the guidance. However, the use of self-selecting samples may not necessarily be representative of the wider population expected to utilise the guidance. Assessment of face validity alone, i.e. a subjective assessment of the relevance of the questions [62] rather than evaluating how well the guidance works when applied prospectively may not identify problems operationalising items. Burford et al. [34] used a prospective design to assess the utility of the reporting guidelines but, largely due to the type of guidance, did not ask reviewers to discuss how they had operationalised the checklist items. In addition, evaluation of the guidance appears to be undertaken by individuals rather than reflecting the collaborative approach that a systematic review encourages. This may be a further reason why the Welch et al. [35] plausibility algorithm had poor inter-rater reliability. Evaluating guidance using a case study approach, may better capture how well the guidance is interpreted or operationalised by those it is designed to assist. Involving novice reviewers in the guidance evaluation may also identify challenges in interpreting and operationalising the guidance that may not necessarily be considered when piloting is undertaken by the guidance developers or expert reviewers alone.

## Strengths and limitations of the review

This study is the first to summarise the range of guidance available on the incorporation of HI in systematic reviews. One of the strengths of this study is detailing what guidance is available for considering HI at various stages of the systematic review process. There is no validated search filter for HI, however, terms were based on those used in a Cochrane methodological review exploring how effects on HI are assessed in systematic reviews [21]. The review did not seek to critique the individual items/questions in the guidance or recommend one guidance over another, but rather offer an overview of guidance available to reviewers when incorporating considerations of HI at different stages of the review process.

A potential limitation of this review is that as this study is part of a PhD study, only one person was involved in the selection of studies, data extraction and synthesis. One limitation of this review is the focus on English language literature when it is acknowledged that other languages, such as Spanish, may offer extensive coverage of literature regarding inequalities. As this is not a systematic review, the search was restricted to a small number of key databases in health, further databases outside of health could have been searched. Instead, a targeted approach to the search was adopted using a number of different search approaches, including scanning of relevant systematic review organisational websites, reference checking and contacting known experts in the field. Given the diverse nature of the guidance documents included in this review, no formal quality appraisal was undertaken instead each guidance document was assessed as to whether or not it formally evaluated.

## Conclusions

Given the recent growing interest in the incorporation of HI in systematic reviews, it is not surprising that methodological guidance exploring how considerations of HI can be incorporated into evidence synthesis is a relatively new and emerging area of research [7, 36]. Above all, the strength of all the guidance documents reviewed in this study is in highlighting the importance of incorporating considerations of HI in systematic reviews, yet due to the fairly recent introduction of the guidance there is little evidence on the guidance uptake or whether it has led to an improvement in considerations of HI in systematic reviews. It is clear, however, that operationalising the guidance will require more work for the reviewer but aside from the Runnels et al. [54] survey, there is limited evidence on the challenges facing reviewers when incorporating considerations of HI. Furthermore, understanding how reviewers can operationalise the guidance and the challenges in doing so have implications not only for understanding the usefulness and burden of the guidance [34], but also has implications for the uptake of guidance and its ultimate goal of improving HI considerations in systematic reviews. There is currently a gap in the evidence examining how reviewers can operationalise the guidance and the barriers and facilitators involved. The results of this review will be used to inform the development of a framework to help reviewers rationalise whether or not to incorporate considerations of HI in systematic reviews.

## Appendix 1: Medline search strategy

### OVID Medline

1 exp Meta-Analysis as Topic/

2 systematic review.tw.

3 meta-analys$.tw.

4 meta-epidemiolog$.tw.

5 exp "Review Literature as Topic"/

6 (Cochrane adj2 review).tw.

7 ((qualitative or realist or evidence or narrative or knowledge) adj synthesis).tw

8 integrative review.tw

9 meta-synthesis.tw

10 meta-ethnography.tw

11 or/1-10

12 (gender-based OR gender-related OR gender differences OR gender factors).tw.

13 ((sex OR gender) adj2 (analysis OR specific OR difference? OR factor? OR inequit$ OR disparit$ OR inequalit$)).tw.

14 exp sex factors/

15 exp geriatrics/

16 ((ethnic$ OR race OR racial OR religio$ OR cultur$ OR minorit$ OR refugee OR indigenous OR aboriginal) adj3 (analysis OR difference$ OR specific OR disparit$ OR inequalit$ OR inequit$)).tw.

17 exp homosexuality/

18 exp disabled persons/

19 ((poverty OR low-income OR socioeconomic$ OR social) adj2 (analysis OR disadvantage$ OR specific OR difference? OR factor? OR inequalit$ OR depriv$ OR inequit$ OR disparit$)).tw.

20 exp Educational Status/

21 exp Socioeconomic Factors/

22 ((discriminat$ OR social exclu$ OR social inclu$) adj3 (religion OR culture OR race OR racial OR aboriginal OR indigenous OR ethnic$)).tw.

23 ((urban OR rural OR inner-city OR slum) adj2 (difference$ OR specific OR analysis OR inequit$ OR disparit$ OR inequalit$)).tw.

24 ((resource-poor OR (low-income adj countr$) OR (middle income adj countr$) OR africa OR developing countr$ OR south america OR china OR asia OR latin america) adj2 (relevance OR analysis OR specific OR difference OR applicab$ OR inequit$ OR disparit$ OR inequalit$)).tw.

25 health adj2 inequalit*.tw

26 health adj2 equit*.tw

27 health adj2 inequit*.tw

28 ((social gradient* or gap) adj3 (reduc* or difference* or disparit* or increase* or inequit* or inequalit* or equit* or disadvantage*)).tw

29 exp Health Status Disparities/

30 or/12-29

31 (guidance or guideline* or tool* or method* or framework* or model*).ti.

32 11 AND 30 AND 31
